# Frequency and predictors of depression and anxiety in chronic illnesses: A multi disease study across non-communicable and communicable diseases

**DOI:** 10.1371/journal.pone.0323126

**Published:** 2025-05-07

**Authors:** Uzair Abbas, Niaz Hussain, Misha Tanveer, Rabeel Nawaz Laghari, Ishfaque Ahmed, Ali Bux Rajper

**Affiliations:** 1 Department of Physiology, Dow University of Health Sciences, Karachi, Pakistan; 2 Bilawal Medical College, Liaquat University of Medical and Health Sciences, Jamshoro, Pakistan; 3 Department of Medicine and Allied, Indus Medical College Hospital, Tando Muhammad Khan, Pakistan,; 4 Department of Infectious Diseases, Sindh Infectious Diseases Hospital and Research Center, DUHS, Karachi, Pakistan; 5 Department of Psychiatry, Bilawal Medical College, Liaquat University of Medical and Health Sciences, Jamshoro, Pakistan; Indian Institute of Information Technology, INDIA

## Abstract

**Background:**

Depression and anxiety are among the most common mental health conditions globally that impact the lifestyle of affected individuals. Mental conditions and chronic diseases are linked to each other bidirectionally. Depression and anxiety with comorbid chronic conditions are often neglected or under-screened and possess challenges in treatment. This study aimed to know the frequency and determinants of depression and anxiety along with the severity level among common chronic communicable and non-communicable diseases.

**Methods:**

We enrolled 200 healthy controls and 800 cases with equal number (n = 400) of patients with communicable and non-communicable diseases. Depression and anxiety were screened through Hamilton’s rating scale for depression and anxiety separately. We also measured the determinants of severe depression among patients with chronic diseases. Data was analyzed through SPSS version 23.

**Results:**

We found higher frequency of depression (31% vs 11%; p=<0.001) and anxiety (13.25% vs 6%; p = 0.021) among cases as compared to healthy controls respectively. We found higher levels of depression among participants with non-communicable diseases as compared to communicable diseases (37.25% vs 24.75%; p < 0.05) respectively. Moreover, there was a higher frequency of anxiety in participants with communicable diseases as compared to those with non-communicable diseases, but the difference was non-significant (14% vs 12.5% p = 0.081). Among non-communicable diseases the highest percentage was found among individuals with cancer (67%), followed by diabetes (38%), cardiovascular diseases (33%) and respiratory disorders (11%). Among participants with communicable diseases, the highest percentage of depression was found in patients with Tuberculosis (29%) followed by HIV/AIDS (28%), Long COVID-19 (25%) and Hepatitis B/C (17%).

**Conclusion:**

There is a significantly higher percentage of depression and anxiety among participants with chronic diseases. It calls for a comprehensive approach to patient care that incorporates mental health as a fundamental aspect of the treatment and management of chronic diseases. Understanding the predictors of severe depression across different chronic conditions helps in stratifying patients who may benefit most from integrated psychiatric and psychological interventions.

## Introduction

Depression and anxiety are among the most common mental health conditions globally [1], affecting individuals across diverse ages, socioeconomic backgrounds, and geographical regions. The Diagnostic and Statistical Manual of Mental Disorders, Fifth Edition (DSM-5) defines depression as a mood disorder characterized by several symptoms that must be present for at least two weeks, such as depressed mood, loss of interest in activities, sleep disturbances, change in appetite etc. While anxiety in DSM-5 is described as a condition characterized by excessive worry and apprehension that occurs more days than not for at least six months. It can also be associated with several other symptoms, including restlessness, fatigue, difficulty in concentrating, irritability, muscle tension, and sleep disturbances [2].

An estimated 3.8% and 4% of the global population suffers from depression and anxiety respectively [3] with different prevalence rates across different regions rising as high as 34% in Pakistan [4]. These disorders contribute significantly to the global burden of disease, leading to impaired daily functioning [5] and substantial economic costs estimated at $382.4 billion [6]. The prevalence of depression and anxiety is not only rising in the general population but is also alarmingly high among individuals with chronic illnesses [7]. Genetic factors, inflammatory pathways, social factors, health behaviors, financial costs and the psychological stress of managing persistent symptoms among patients with chronic diseases contribute to this heightened vulnerability [7].

In high income countries, severity of anxiety and depressive symptoms was strongly associated with having chronic medical conditions such as diabetes, pulmonary disease, heart disease, and arthritis [7,8]. Studies indicated a prevalence of depression among in-patients was 12% [9] and in out-patients was 27.0% [10]. Chronic diseases and mental health disorders such as depression and anxiety are interconnected through shared inflammatory pathways that significantly impact both physical and psychological well-being [11]. Pro-inflammatory cytokines affect critical biological systems associated with the pathophysiology of depression. Raised IL-1, IL-6, and TNF-alpha which are common in chronic conditions such as diabetes, have bidirectional relationship, affecting neurotransmitter functions and mood regulation. In addition, administration of proinflammatory cytokines in cancer or hepatitis C therapies, has been found to induce depressive symptomatology [11].

Pakistan is facing a double burden of diseases, with communicable diseases (CD) at 40% [12] and non-communicable diseases (NCD) at 58% [13] which is a huge challenge. However, the prevalence of depression and anxiety remains underexplored, particularly in the context of chronic illnesses. Limited studies in the region suggest a moderately high prevalence of anxiety and depressive symptoms among adults with multimorbidity aged 30 years and above [14].

The present study seeks to provide an updated estimation of the prevalence of depression and anxiety among adults in Karachi, Pakistan, with a specific focus on individuals living with chronic illnesses. We have selected NCD’s such as Diabetes, CVD, Respiratory diseases, Cancers, and CD’s such as Tuberculosis, HIV/AIDS, Long COVID-19, Hepatitis B, and C based on their high prevalence and significant impact on public health in Pakistan. This research aims to generate actionable data to inform targeted public health strategies and clinical interventions to enhance patient outcomes, reduce healthcare costs, and foster more effective, integrated models of care for managing both mental and physical health conditions.

## Methods

### Ethical consideration

The study was conducted as per ethical guidelines of declaration of Helsinki. It was approved by the Institutional Review Board (IRB) of Dow University of Health Sciences (DUHS). The IRB approval reference number is IRB-2342/DUHS/Approval/2022–652. The participants were recruited in the study after written informed consent.

### Study Description and setting

In this cross-sectional, we included a total of 1000 age and gender matched participants from Dow University Hospital (DUH), Karachi Pakistan from January 2024 to November 2024. The sample size was calculated from OpenEPI sample size calculator. With estimated prevalence of depression and anxiety 30% (±5%) and design effect (DEFF) of 3, the sample size appeared to be 969. However, we included a total of 1000 individuals in our study.

### Recruitment of participants

#### Cases.

A total of 800 participants with age 20–60 years, female or male were included in the study after taking written informed consent. This group was categorized into two main sub-groups as follows.

**I.Non-communicable disease group:** These were 400 individuals with non-communicable diseases diagnosed with either Diabetes mellitus, Cardiovascular disorders (hypertension and chronic artery disease), respiratory disorders (Asthma, COPD) and cancers (lung, prostate, breast and colon). These individuals were previously diagnosed by physicians and recruited from the respective outpatients’ departments (OPDs) with each group having 100 participants matched for demographic characteristics.

**II.Communicable disease group:** There were 400 individuals with communicable diseases diagnosed with tuberculosis, HIV/AIDS, Long COVID-19 or hepatitis B/C with each group having 100 participants. These individuals were previously diagnosed by physicians and recruited from respective OPDs after matching demographic characteristics.

Admitted patients and those who were already diagnosed with depression and anxiety were excluded from the study. Those who were prescribed for any anti-depressive or anti-anxiety medicines in past were also excluded from the study.

#### Healthy (HC) group.

We included 200 healthy individuals who had none of the above diagnosed diseases. Healthy individuals were of age 20–60 years, either male or female genders. The participants of healthy group were attendants of the patients with the same exclusion criteria. The medical, para medical staff and medical students were excluded from the study.

#### Data collection.

In all participants, at the time of recruitment, demographic data regarding age, gender, marital status, monthly income, and level of education (literate or illiterate) was recorded. The clinical data was obtained separately for each disease type.

#### Assessment of depression and anxiety.

Hamilton Depression Rating Scale was administered to assess the presence of depression and its severity. The scale consists of 17 items and score ranges from 0 to 54 where 0–7 indicate no depression, 8–13 indicate mild depression, 14–18 indicate moderate depression and ≥ 19 indicate severe depression. The scale has been used to assess depression and its severity among multiple medical conditions [15].

Hamilton Anxiety Rating Scale was used to assess the presence of anxiety. This scale consists of 14 items with a total score range of 0–56, where 0–5 indicate no anxiety, 6–14 indicate mild anxiety, 15–28 indicate moderate anxiety and ≥ 29 indicate severe anxiety. This scale has also been used to assess anxiety and its severity among multiple medical conditions [16].

#### Data analysis.

Statistical analysis was carried out using the Statistical Packages for Social Sciences (SPSS) version 23. Mean and frequencies were calculated through simple statistical analysis. The Pearson Chi square test was used to compare categorical data. While the Mann-Witney T test was used to compare the median values. Multiple regression was used to know the predictors of severe depression and anxiety in each disease type. P values <0.05 with 95% CI were considered as significant.

## Results

### a. Demographic characteristics of study participants

We included 1000 individuals in our study. Out of those, 200 were healthy controls and 800 were patients with either non-communicable diseases (n = 400) or chronic infectious diseases (n = 400). There was no significant difference in age and gender between cases and controls (p > 0.05). We found a significant higher frequency of depression (248/800;31% vs 22/200;11%; p=<0.001) and anxiety (106/800;13.25% vs 12/200;6%; p = 0.021) among cases as compared to healthy controls respectively. The demographic characteristics of cases and healthy controls are compared in Table 1.

**Table 1:** Demographic characteristics of study participants.

Item	Variables	Total (n = 1000)	Healthy controls (n = 200)	Cases (n = 800)	*p* value (cases vs controls)
Age	Mean (years)	47.01 ± 11.23	49.87 ± 9.19	52.65 ± 7.27	0.37^#^
Gender	Female	487	97 (48.5%)	390 (48.75%)	0.99
	Male	513	103 (51.5%)	410 (51.25%)	
					
Marital status	Married	710	144 (72%)	566 (70.75%)	0.96
	Unmarried	290	56 (28%)	234 (29.25%)	
					
Level of education	Literate**	684	127 (63.5%)	557 (69.62%)	0.63
					
Level of income (PKR/month)	0-50k	213	47 (23.5%)	166 (20.75%)	0.87
	51k-100k	287	57 (28.5%)	230 (28.75%)	
	101k-200k	244	48 (24%)	196 (24.5%)	
	200k above	256	48 (24%)	208 (26%)	
					
Frequency of depression		270	22 (11%)	248 (31%)	<0.001*
Frequency of anxiety		118	12 (6%)	106 (13.25%)	0.021*
					

The table shows demographic characteristics and frequency of depression and anxiety among study participants. ^#^Compared through T test. *Shows significant p value calculated from Pearson Chi square. ** Literate were defined as those who completed middle school or higher.

### b. Frequency of depression and anxiety in communicable and non-communicable diseases

**Fig 1:**
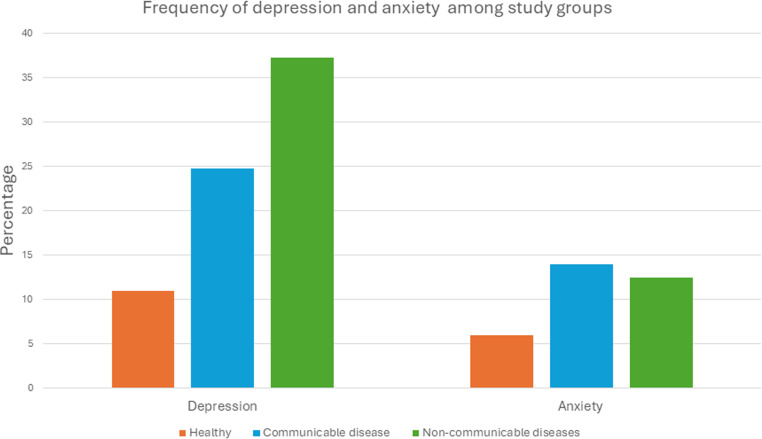
Frequency of depression and anxiety among study participants (n = 1000). The figure shows the distribution of anxiety and depression among healthy controls (n = 200), participants with communicable (n = 400) and non-communicable diseases (n = 400).

We further explored the differential frequency of depression and anxiety among different groups in our study. We found higher levels of depression among participants with non-communicable diseases as compared to communicable diseases (149/400; 37.25% vs 99/400;24.75%; p < 0.05) respectively. However, there was a higher frequency of anxiety in participants with communicable diseases as compared to those with non-communicable diseases, but the difference was non-significant (14% vs 12.5% p = 0.081; Fig 1).

### c. Disease wise distribution of depression among study participants

**Fig 2:**
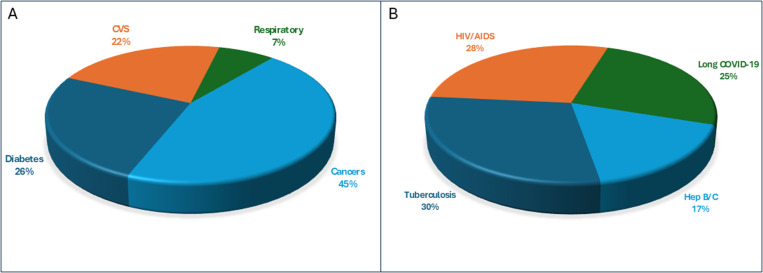
Disease wise distribution of depression among study participants. A, non-communicable diseases (n = 149). B, communicable diseases (n = 99).

We compared the frequency of depression among individuals with different chronic disorders among communicable and non-communicable diseases. Among non-communicable diseases we found a higher percentage (38/100; 38%) of depression among diabetic patients followed by cardiovascular diseases (33/100; 33%), and respiratory disorders (11/100; 11%). The highest percentage of depression was found among individuals with cancers (67/100; 67%). The percentage distribution of depression among non-communicable diseases is given in Fig 2A.

Among participants with communicable diseases, we found the highest percentage of depression among individuals with Tuberculosis (29/100; 29%) followed by HIV/AIDS (28/100; 28%), Long COVID-19 (25/100; 25%) and Hepatitis B/C (17/100; 17%). The percentage distribution of depression among communicable diseases is given in Fig 2B.

### d. Disease wise distribution of anxiety among study participants

**Fig 3:**
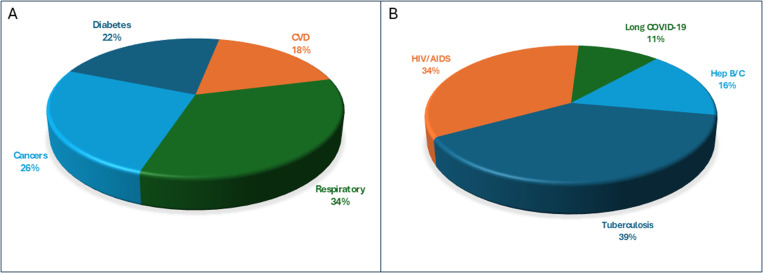
Disease wise distribution of anxiety among study participants. A, non-communicable diseases (n = 40). B, communicable diseases (n = 56).

We also compared the frequency of anxiety among individuals with different chronic disorders among communicable and non-communicable diseases. Among non-communicable diseases, we found 11% (11/100) of anxiety among diabetic patients, and 9% (9/100) among patients with CVD. Individuals with respiratory disorders were found to have the highest percentage (17/100; 17%) of anxiety while among individuals with cancers, we found 13% (13/100). The percentage distribution of depression among non-communicable diseases is given in Fig 3A.

Among participants with communicable diseases, we found the highest percentage of anxiety among individuals with Tuberculosis (22/100; 22%) followed by HIV/AIDS (19/100; 19%), Hepatitis B/C (9/100; 9%) and Long COVID-19 (6/100; 6%). The percentage of depression among communicable diseases is given in Fig 3B.

### e. Association of severity of depression with non-communicable and communicable diseases

We further compared the severity of depression among participants with respect to communicable and non-communicable diseases. Among patients with non-communicable diseases, we found a total of 39 (26.17%) individuals with mild and 44 individuals (29.5%) with moderate depression. However, 66/149 (44.2%) were found to have severe depression (p = 0.0032). There were significantly higher cases of severe depression among participants with diabetes, cardiovascular disorders, cancers, tuberculosis and HIV/AIDS (p < 0.05; Table 2). No difference in severity of depression was reported among patients with respiratory disorders, Long COVID-19 and Hepatitis B/C (p value >0.05; Table 2).

**Table 2:** Association of severity of depression with non-communicable and communicable diseases.

Disease type		Severity of depression (Hamilton’s scale)			*p* value
		Mild (8–13)	Moderate (14–18)	Severe (≥19)	
Non-communicable diseases (n = 149)	Diabetes (n = 38)	9 (23.68%)	13 (34.21%)	16 (42.1%)	0.013*
	CVD (n = 33)	10 (30.3%)	08 (24.24%)	15 (45.45%)	0.024*
	Respiratory (n = 11)	04 (36.36%)	04 (36.36%)	03 (27.27%)	0.371
	Cancers (n = 67)	16 (23.88%)	19 (28.36%)	32 (47.76%)	0.002*

Communicable diseases (n = 99)	Tuberculosis (n = 29)	8 (27.58%)	7 (24.13%)	14 (48.27%)	0.012*
	HIV/AIDS (n = 28)	4 (14.28%)	6 (21.42%)	18 (64.28%)	0.004*
	Long COVID-19 (n = 25)	11 (44%)	8 (32%)	6 (24%)	0.058
	Hepatitis B/C (n = 17)	6 (35.29%)	5 (29.41%)	6 (35.29%)	0.873

The table shows the association of severity of depression with type of disease among study participants. Percentages were calculated in rows. *Shows significant p value calculated from Pearson Chi square.

### f. Association of severity of anxiety with non-communicable and communicable diseases

Moving further, we assessed the severity of anxiety among participants with respect to communicable or non-communicable diseases. We found a total of 17 (34%) individuals with mild and 18 individuals (36%) with moderate anxiety. However, 15/50 (30%) were found to have severe anxiety among patients with non-communicable diseases (p = 0.29). We found significantly least mild cases of anxiety among HIV/AIDS patients (p = 0.03) while no significant association found in severity of anxiety with type of disease (p > 0.05; Table 3).

**Table 3:** Association of severity of anxiety with non-communicable and communicable diseases.

Disease type		Severity of anxiety (Hamilton’s scale)			*p* value
		Mild (6–14)	Moderate (15–28)	Severe (≥29)	
Non-communicable diseases (n = 50)	Diabetes (n = 11)	3 (27.27%)	5 (45.45%)	3 (27.27%)	0.09
	CVD (n = 9)	4 (44.44%)	3 (33.33%)	2 (22.22%)	0.13
	Respiratory (n = 17)	6 (35.29%)	5 (29.41%)	6 (35.29%)	0.89
	Cancers (n = 13)	4 (30.76%)	5 (38.46%)	4 (30.76%)	0.76

Communicable diseases (n = 56)	Tuberculosis (n = 22)	6 (27.27%)	7 (31.81%)	9 (40.9%)	0.38
	HIV/AIDS (n = 19)	11 (57.89%)	6 (31.57%)	2 (10.52%)	0.03*
	Long COVID-19 (n = 6)	3 (50%)	3 (50%)	0	0.99
	Hepatitis B/C (n = 9)	4 (44.44%)	2 (22.22%)	3 (33.33%)	0.16

The table shows the association of severity of anxiety with type of disease among study participants. Percentages were calculated in rows. *Shows significant p value calculated from Pearson Chi square.

### g. Predictors of severe depression among non-communicable and communicable diseases

We further explored the predictors of severe depression among participants with non-communicable and communicable diseases. Among patients with non-communicable diseases, in participants who had diabetes, we found increased age (OR = 1.932; p = 0.012), married (OR = 1.12; p = 0.001), being on insulin therapy (OR = 5.13; p = 0.004), high HbA1c (OR = 6.98; p = 0.001), more years since diagnosis of DM (OR = 4.56; p = 0.001) and presence of one or more diabetes related complications (OR = 5.512; p = 0.001) were strong predictors of having severe depression. For cardiovascular diseases, having male gender (OR = 2.19; p = 0.022), married (OR = 0.9; p = 0.011), hypertension (OR = 3.78; p = 0.032), and more years of diagnosis (OR = 2.13; p = 0.0043) were found to be predictors of severe depression. Moreover, male gender (OR = 2.31; p = 0.0021), having lung cancer (OR = 3.15; p = 0.0031), being on radiotherapy or chemotherapy (OR = 6.13; p = 0.001) were strong predictors of severe depression among cancer patients.

While exploring predictors of severe depression among patients with chronic communicable diseases, we found that increased age (OR = 1.98; p = 0.004); male gender (OR = 2.39; p = 0.014), having MDR/XDR TB (OR = 3.12; p = 0.001), having diabetes as comorbid (OR = 4.72; p = 0.001) were predictors of severe depression among tuberculosis patients. Participants with long COVID-19, we found female gender (OR = 1.78; p = 0.004), having more than 2 symptoms of long COVID-19 (OR = 4.38; p = 0.001) were predictors of severe depression among them.

## Discussion

This study highlights the significant mental health burden among individuals with chronic diseases, demonstrating a contrast in the prevalence of depression and anxiety between those with chronic conditions and healthy controls. The frequency of depression was significantly higher among cases than controls (31% vs. 11%, p < 0.001), and a similar trend was observed for anxiety (13.25% vs. 6%, p = 0.021). These findings are consistent with previous studies which suggest that chronic disease conditions are associated with increased rates of mental health disorders due to the stress and lifestyle limitations they impose on individuals [14,17,18-19].

We found that depression was significantly more prevalent among patients with NCDs (37.25%) compared to those with CDs (24.75%). This could be attributed to the long-term, progressive nature of NCDs, which often come with debilitating symptoms, a decline in quality of life, and decrease in life expectancy [20,21]. Chronic management of diseases such as diabetes, cardiovascular diseases, and cancer can also induce significant psychological stress, leading to depression [22].

While the prevalence of anxiety was higher among individuals with CDs as compared to NCD patients (14% vs. 12.5%), however the difference was not statistically significant. This observation may reflect the acute stress and uncertainty caused by social isolation associated with infectious diseases, which can trigger anxiety [23].

Our study’s findings illustrate a distinct distribution of depression and anxiety across various chronic diseases, both communicable and non-communicable. Notably, NCD’s such as cancer exhibited the highest rates of depression (67%), a statistic that aligns with previous studies emphasizing the severe psychological impact of cancer diagnoses and treatments [24,25]. These treatments often involve prolonged suffering and uncertain prognoses, which can significantly deteriorate mental health. Similarly, diabetes and cardiovascular diseases showed substantial depression rates of 38% and 33%, respectively. The chronic nature of these conditions, coupled with the burden of ongoing management and potential complications, contributes to these elevated levels of depression [26,27]. A study on Pakistani population gave a strong correlation between depression and chronic diseases especially anemia and diabetes [28].

For CDs, tuberculosis and HIV/AIDS were notable for their high depression rates, at 29% and 28% respectively. The fear of contagion and the complexity of their treatment regimens can exacerbate mental health issues, reflecting findings from other studies that highlighted similar psychological burdens among these patient groups [29–31].

Mental health treatment regimens and the management of chronic diseases often interact in complex ways that can complicate treatment approaches, presenting additional challenges for holistic management. For example, Selective Serotonin Reuptake Inhibitors (SSRIs) used for depression can interact with anticoagulants used in cardiovascular disease, increasing the risk of bleeding [32]. Anti-tuberculosis drugs particularly isoniazid and linezolid significantly interact with anti-depressants, others may cause adverse psychiatric effects, and drugs like rifampicin can lower the efficacy of antipsychotics due to their enzyme-inducing properties further exacerbating psychiatric issues and complicating treatment [33]. Concurrent use of SSRIs can inhibit specific CYP450 enzymes, impacting the metabolism of some chemotherapeutic agents like tamoxifen [34].

Concerning anxiety, our findings indicate that respiratory disorders lead among non-communicable diseases with a 17% rate, likely due to the acute and chronic stress associated with breathing difficulties and the direct impact on physical autonomy [35]. Cancer patients also showed significant anxiety levels (13%), which may be influenced by the life-threatening nature of the disease and the intense treatment regimen involved.

Among communicable diseases, tuberculosis had the highest anxiety rates (22%), followed by HIV/AIDS (19%), Hepatitis B/C (9%) and Long COVID-19 (6%), which might be reinforced by the prolonged and isolating treatment required, adding to the existing literature that stresses the psychological effects of TB treatment [36]. Diseases such as tuberculosis and HIV/AIDS not only involve complex treatment regimens but also carry social stigmas that may contribute to heightened anxiety levels as supported by studies on the psychosocial impacts of infectious diseases like HIV/AIDS and tuberculosis [37–40].

Our analysis focused on identifying predictors of severe depression among individuals with chronic diseases. Among patients with NCDs, several key predictors of severe depression were identified. In diabetes advanced age, marital status, the requirement for insulin therapy, high HbA1c levels, longer disease duration, and the presence of complications were significantly associated with severe depression. These findings suggest that the burden of diabetes management, particularly with complications and poor glycemic control, may contribute to psychological distress. The increase in pro-inflammatory mediators can be a plausible link explaining the increased severity of depression among patients with glycemic control as supported by various studies [41–44].

For CVD being male, married, having hypertension, and a longer duration since diagnosis were predictors of severe depression. This might reflect the stress associated with managing long-term, life-threatening conditions which can adversely affect prognosis as evidenced in existing literature [45]. In patients with cancer, the male gender, undergoing chemotherapy or radiotherapy, and having lung cancer were strongly linked to higher rates of severe depression. A review article documented that, similar to diabetic pathophysiology, cancer occurrence and anticancer treatments also results in the pro-inflammatory cytokines-mediated inflammation, which dysregulates the HPA-axis activity that may result in depression-like behavior. Conversely, depression can also trigger HPA-axis activation that results in the downstream release of endogenous glucocorticoids which may cause depressive symptomatology [46]. The psychological impact of cancer diagnosis and treatment is well recognized [25,47]; however, our findings highlight the particularly intense impact of lung cancer and aggressive treatments, which may be due to the poor prognosis and high symptom burden associated with these conditions.

In the realm of communicable diseases, notable factors for TB were increased age, male gender, having multi-drug-resistant TB (MDR/XDR TB), and the presence of diabetes as a comorbidity were identified as predictors of severe depression. Interestingly, in long COVID-19 patients, factors such as being female and experiencing more than two symptoms of long COVID-19 were linked to severe depression. This could be due to the ongoing and uncertain nature of long COVID-19, which can lead to significant anxiety and stress, contributing to depressive symptoms

These findings underscore the necessity for healthcare providers to adopt a holistic approach in managing patients with chronic diseases, incorporating routine mental health assessments, especially for those identified at high risk for severe depression. For patients with diabetes, cardiovascular diseases, and cancer, regular screening for depression and tailored psychological interventions should be considered as part of standard care protocols. Similarly, for communicable diseases like TB and long COVID-19, addressing mental health needs is crucial, particularly considering the stigma and long-term symptoms associated with these conditions.

## Conclusion

This study adds to the growing body of evidence that chronic physical health conditions are a significant predictor of mental health problems, particularly depression and anxiety. It calls for a comprehensive approach to patient care that incorporates mental health as a fundamental aspect of the treatment and management of chronic diseases. Understanding the predictors of severe depression across different chronic conditions helps in stratifying patients who may benefit most from integrated psychiatric and psychological interventions. This approach not only aims to improve quality of life but also enhances the effectiveness of disease management programs, ultimately reducing the overall healthcare burden.

## References

[1] Evaluation IoHMa. Global Burden of Disease Results Tool: Institute for Health Metrics and Evaluation. University of Washington; 2024. Available from: https://vizhub.healthdata.org/gbd-results/.

[2] Williams JB, First M. Diagnostic and statistical manual of mental disorders. Encyclopedia of Social Work. 2013.

[3] WHO. Depressive disorder (depression). World Health Organization; 2023. Available from: https://www.who.int/news-room/fact-sheets/detail/depression

[4] Mirza I, Jenkins R. Risk factors, prevalence, and treatment of anxiety and depressive disorders in Pakistan: systematic review. BMJ. 2004;328(7443):794. doi: 10.1136/bmj.328.7443.794 15070634 PMC383372

[5] GBD 2019 Mental Disorders Collaborators. Global, regional, and national burden of 12 mental disorders in 204 countries and territories, 1990-2019: a systematic analysis for the Global Burden of Disease Study 2019. Lancet Psychiatry. 2022;9(2):137–50. doi: 10.1016/S2215-0366(21)00395-3 35026139 PMC8776563

[6] Greenberg P, Chitnis A, Louie D, Suthoff E, Chen S-Y, Maitland J, et al. The Economic Burden of Adults with Major Depressive Disorder in the United States (2019). Advances in Therapy. 2023;40(10):4460–79. 37518849 10.1007/s12325-023-02622-x PMC10499687

[7] Niles AN, Dour HJ, Stanton AL, Roy-Byrne PP, Stein MB, Sullivan G, et al. Anxiety and depressive symptoms and medical illness among adults with anxiety disorders. J Psychosom Res. 2015;78(2):109–15. 25510186 10.1016/j.jpsychores.2014.11.018 PMC4297513

[8] Katon W, Lin EHB, Kroenke K. The association of depression and anxiety with medical symptom burden in patients with chronic medical illness. Gen Hosp Psychiatry. 2007;29(2):147–55. doi: 10.1016/j.genhosppsych.2006.11.005 17336664

[9] Walker J, Burke K, Wanat M, Fisher R, Fielding J, Mulick A, et al. The prevalence of depression in general hospital inpatients: a systematic review and meta-analysis of interview-based studies. Psychol Med. 2018;48(14):2285–98. doi: 10.1017/S0033291718000624 29576041

[10] Wang J, Wu X, Lai W, Long E, Zhang X, Li W, et al. Prevalence of depression and depressive symptoms among outpatients: a systematic review and meta-analysis. BMJ Open. 2017;7(8):e017173. doi: 10.1136/bmjopen-2017-017173 28838903 PMC5640125

[11] Schiepers OJG, Wichers MC, Maes M. Cytokines and major depression. Prog Neuropsychopharmacol Biol Psychiatry. 2005;29(2):201–17. doi: 10.1016/j.pnpbp.2004.11.003 15694227

[12] Bilal W, Qamar K, Abbas S, Siddiqui A, Essar MY. Infectious diseases surveillance in Pakistan: Challenges, efforts, and recommendations. Ann Med Surg (Lond). 2022;78:103838. doi: 10.1016/j.amsu.2022.103838 35734665 PMC9207098

[13] Kazmi T, Nagi M, Razzaq S, Hussnain S, Shahid N, Athar U. Burden of noncommunicable diseases in Pakistan. East Mediterr Health J. 2022;28(11):798–804. doi: 10.26719/emhj.22.083 36515443

[14] Farooq S, Khan T, Zaheer S, Shafique K. Prevalence of anxiety and depressive symptoms and their association with multimorbidity and demographic factors: a community-based, cross-sectional survey in Karachi, Pakistan. BMJ Open. 2019;9(11):e029315. doi: 10.1136/bmjopen-2019-029315 31748286 PMC6887067

[15] Bagby RM, Ryder AG, Schuller DR, Marshall MB. The Hamilton Depression Rating Scale: has the gold standard become a lead weight? Am J Psychiatry. 2004;161(12):2163–77. doi: 10.1176/appi.ajp.161.12.2163 15569884

[16] Thompson E. Hamilton Rating Scale for Anxiety (HAM-A). Occup Med (Lond). 2015;65(7):601. doi: 10.1093/occmed/kqv054 26370845

[17] Gunn JM, Ayton DR, Densley K, Pallant JF, Chondros P, Herrman HE, et al. The association between chronic illness, multimorbidity and depressive symptoms in an Australian primary care cohort. Soc Psychiatry Psychiatr Epidemiol. 2012;47(2):175–84. doi: 10.1007/s00127-010-0330-z 21184214

[18] Winkler P, Horáček J, Weissová A, Šustr M, Brunovský M. Physical Comorbidities in Depression Co-Occurring with Anxiety: A Cross Sectional Study in the Czech Primary Care System. Int J Environ Res Public Health. 2015;12(12):15728–38. doi: 10.3390/ijerph121215015 26690458 PMC4690951

[19] O’Connor K, Vizcaino M, Ibarra JM, Balcazar H, Perez E, Flores L, et al. Multimorbidity in a Mexican Community: Secondary Analysis of Chronic Illness and Depression Outcomes. Int J Nurs (N Y). 2015;2(1):35–47. 26640817 10.15640/ijn.v2n1a4 PMC4667743

[20] Cimpean D, Drake RE. Treating co-morbid chronic medical conditions and anxiety/depression. Epidemiol Psychiatr Sci. 2011;20(2):141–50. doi: 10.1017/s2045796011000345 21714361

[21] Evangelista JMV, Soares V, Mendanha LPM, Oliveira-Silva I, Lima WA, Ribeiro HL, et al. Depression and anxiety in subjects with chronic non‑communicable diseases. Manual Therapy. Posturology & Rehabilitation Journal. 2017:1–5.

[22] Anwar N, Kuppili PP, Balhara YPS. Depression and physical noncommunicable diseases: The need for an integrated approach. WHO South East Asia J Public Health. 2017;6(1):12–7. doi: 10.4103/2224-3151.206158 28597853

[23] Chew QH, Wei KC, Vasoo S, Chua HC, Sim K. Narrative synthesis of psychological and coping responses towards emerging infectious disease outbreaks in the general population: practical considerations for the COVID-19 pandemic. Singapore Med J. 2020;61(7):350–6. doi: 10.11622/smedj.2020046 32241071 PMC7926608

[24] Liu M, Yan R, Lu S, Zhang P, Xu S. Pathogenesis and therapeutic strategies for cancer-related depression. Am J Cancer Res. 2024;14(9):4197–217. doi: 10.62347/WVVG5364 39417166 PMC11477823

[25] Riedl D, Schüßler G. Factors associated with and risk factors for depression in cancer patients - A systematic literature review. Transl Oncol. 2022;16:101328. doi: 10.1016/j.tranon.2021.101328 34990907 PMC8741617

[26] Namisango E, Powell RA, Taylor S, Radbruch L, Freeman R, Haufiku D, et al. Depressive Symptoms and Palliative Care Concerns Among Patients With Non-communicable Diseases in Two Southern African Countries. J Pain Symptom Manage. 2023;65(1):26–37. doi: 10.1016/j.jpainsymman.2022.09.008 36162705

[27] Armbrecht E, Shah R, Poorman GW, Luo L, Stephens JM, Li B, et al. Economic and Humanistic Burden Associated with Depression and Anxiety Among Adults with Non-Communicable Chronic Diseases (NCCDs) in the United States. J Multidiscip Healthc. 2021;14:887–96. doi: 10.2147/JMDH.S280200 33935498 PMC8079356

[28] Godil A, Mallick MSA, Adam AM, Haq A, Khetpal A, Afzal R, et al. Prevalence and Severity of Depression in a Pakistani Population with at least One Major Chronic Disease. J Clin Diagn Res. 2017;11(8):OC05–OC10. doi: 10.7860/JCDR/2017/27519.10329 28969176 PMC5620817

[29] Redwood L, Mitchell EMH, Viney K, Snow K, Nguyen TA, Dung LAT, et al. Depression, stigma and quality of life in people with drug-susceptible TB and drug-resistant TB in Vietnam. Int J Tuberc Lung Dis. 2021;25(6):461–7. doi: 10.5588/ijtld.20.0952 34049608

[30] Chauhan A, Pandya A, Bhatt D, Salaliya V, Trivedi R, Kapadia D, et al. Dual burden of TB and mental ill-health: Prevalence and associated factors of anxiety and depression among TB patients in Gujarat. J Family Med Prim Care. 2024;13(12):5615–20. doi: 10.4103/jfmpc.jfmpc_532_24 39790810 PMC11709073

[31] MacLean JR, Wetherall K. The Association between HIV-Stigma and Depressive Symptoms among People Living with HIV/AIDS: A Systematic Review of Studies Conducted in South Africa. J Affect Disord. 2021;287:125–37. doi: 10.1016/j.jad.2021.03.027 33780828

[32] Rahman AA, He N, Rej S, Platt RW, Renoux C. Concomitant Use of Selective Serotonin Reuptake Inhibitors and Oral Anticoagulants and Risk of Major Bleeding: A Systematic Review and Meta-analysis. Thromb Haemost. 2023;123(1):54–63. doi: 10.1055/a-1932-8976 36037829

[33] Doherty AM, Kelly J, McDonald C, O’Dywer AM, Keane J, Cooney J. A review of the interplay between tuberculosis and mental health. Gen Hosp Psychiatry. 2013;35(4):398–406. doi: 10.1016/j.genhosppsych.2013.03.018 23660587

[34] Singh MS, Francis PA, Michael M. Tamoxifen, cytochrome P450 genes and breast cancer clinical outcomes. Breast. 2011;20(2):111–8. doi: 10.1016/j.breast.2010.11.003 21185724

[35] Hurtado-Ruzza R, Iglesias ÓÁ-C, Dacal-Quintas R, Becerro-de-Bengoa-Vallejo R, Calvo-Lobo C, San-Antolín M, et al. Asthma, much more than a respiratory disease: influence of depression and anxiety. Rev Assoc Med Bras (1992). 2021;67(4):571–6. doi: 10.1590/1806-9282.20201066 34495063

[36] Agbeko CK, Mallah MA, He B, Liu Q, Song H, Wang J. Mental Health Status and Its Impact on TB Treatment and Its Outcomes: A Scoping Literature Review. Front Public Health. 2022;10:855515. doi: 10.3389/fpubh.2022.855515 35712316 PMC9194388

[37] Gooden TE, Gardner M, Wang J, Chandan JS, Beane A, Haniffa R, et al. The risk of mental illness in people living with HIV in the UK: a propensity score-matched cohort study. Lancet HIV. 2022;9(3):e172–81. doi: 10.1016/S2352-3018(21)00319-2 35123667

[38] Olashore AA, Paruk S, Akanni OO, Tomita A, Chiliza B. Psychiatric Disorders in Adolescents Living with HIV and Association with Antiretroviral Therapy Adherence in Sub-Saharan Africa: A Systematic Review and Meta-analysis. AIDS Behav. 2021;25(6):1711–28. doi: 10.1007/s10461-020-03100-z 33216245

[39] Hayward SE, Deal A, Rustage K, Nellums LB, Sweetland AC, Boccia D, et al. The relationship between mental health and risk of active tuberculosis: a systematic review. BMJ Open. 2022;12(1):e048945. doi: 10.1136/bmjopen-2021-048945 34992103 PMC8739435

[40] Febi AR, Manu MK, Mohapatra AK, Praharaj SK, Guddattu V. Psychological stress and health-related quality of life among tuberculosis patients: a prospective cohort study. ERJ Open Res. 2021;7(3):00251–2021. doi: 10.1183/23120541.00251-2021 34476253 PMC8405877

[41] Stuart MJ, Baune BT. Depression and type 2 diabetes: inflammatory mechanisms of a psychoneuroendocrine co-morbidity. Neurosci Biobehav Rev. 2012;36(1):658–76. doi: 10.1016/j.neubiorev.2011.10.001 22020230

[42] Jeon SW, Kim YK. Neuroinflammation and cytokine abnormality in major depression: Cause or consequence in that illness?. World J Psychiatry. 2016;6(3):283–93. doi: 10.5498/wjp.v6.i3.283 27679767 PMC5031928

[43] Réus GZ, Dos Santos MAB, Strassi AP, Abelaira HM, Ceretta LB, Quevedo J. Pathophysiological mechanisms involved in the relationship between diabetes and major depressive disorder. Life Sci. 2017;183:78–82. doi: 10.1016/j.lfs.2017.06.025 28676432

[44] Rustad JK, Musselman DL, Nemeroff CB. The relationship of depression and diabetes: pathophysiological and treatment implications. Psychoneuroendocrinology. 2011;36(9):1276–86. doi: 10.1016/j.psyneuen.2011.03.005 21474250

[45] Mensah GA, Collins PY. Understanding mental health for the prevention and control of cardiovascular diseases. Glob Heart. 2015;10(3):221–4. doi: 10.1016/j.gheart.2015.08.003 26407518 PMC4584120

[46] Ahmad MH, Rizvi MA, Fatima M, Mondal AC. Pathophysiological implications of neuroinflammation mediated HPA axis dysregulation in the prognosis of cancer and depression. Mol Cell Endocrinol. 2021;520:111093. doi: 10.1016/j.mce.2020.111093 33253761

[47] Naser AY, Hameed AN, Mustafa N, Alwafi H, Dahmash EZ, Alyami HS, et al. Depression and Anxiety in Patients With Cancer: A Cross-Sectional Study. Front Psychol. 2021;12:585534. doi: 10.3389/fpsyg.2021.585534 33935849 PMC8081978

